# Integrated AI-Driven Discovery of MAPK3 Inhibitors for Oral Inflammatory and Proliferative Diseases

**DOI:** 10.3390/ph19081309

**Published:** 2026-08-19

**Authors:** Muhammad Ishfaq, Shahi Jahan Shah, Imran Khalid, Mashail M. M. Hamid, Muhammad Zahir Kota, Abdul Ahad Ghaffar Khan, Mohammed Ibrahim, Samuel Ebele Udeabor, Abosofyan Salih Atta Elfadeel Mohamed Salih, Chidozie Ifechi Onwuka

**Affiliations:** Department of Oral and Maxillofacial Surgery, College of Dentistry, King Khalid University, Abha 62521, Saudi Arabia

**Keywords:** MAPK3, oral squamous cell carcinoma, machine learning QSAR, molecular docking, molecular dynamics simulation

## Abstract

**Background**: Mitogen-activated protein kinase 3 (MAPK3/ERK1) plays a central role in cellular proliferation, inflammation, apoptosis, and survival signalling and has been implicated in oral squamous cell carcinoma (OSCC), periodontitis, oral lichen planus, and other chronic oral inflammatory diseases. The present study employed an integrated computational workflow combining machine learning (ML)-based quantitative structure–activity relationship (QSAR) modelling, molecular docking, density functional theory (DFT), molecular dynamics (MD) simulation, and MM-GBSA analysis to identify and characterise potent MAPK3 inhibitors. **Methods**: A curated dataset of 907 experimentally validated MAPK3 inhibitors was retrieved from the ChEMBL database and processed using molecular descriptors and Morgan fingerprints. Multiple ML algorithms were evaluated under scaffold-based validation, with Light Gradient Boosting Machine (LightGBM) demonstrating the best predictive performance. **Results**: The final model achieved strong classification capability with ROC-AUC values of 0.898 and 0.926. Feature importance analysis revealed that local structural motifs captured by fingerprint descriptors played dominant roles in MAPK3 inhibitory activity. The top-ranked compounds were subjected to molecular docking, where compounds 58324148 and 137531515 exhibited strong binding affinities of −11.9 and −11.0 kcal/mol, respectively. DFT calculations demonstrated favourable electronic properties with low HOMO–LUMO energy gaps, while MD simulations confirmed stable receptor–ligand interactions throughout 200 ns trajectories. MM-GBSA analysis further supported strong binding stability dominated by van der Waals interactions. **Conclusions**: Overall, the integrated computational framework successfully identified promising MAPK3 inhibitor candidates with potential therapeutic relevance for oral inflammatory and proliferative diseases.

## 1. Introduction

The mitogen-activated protein kinases (MAPKs) are a highly conserved family of protein kinases involved in the regulation of essential cell functions such as proliferation, differentiation, apoptosis, inflammation, stress response, and survival signalling [1,2,3,4]. Mitogen-activated protein kinase 3, or extracellular signal-regulated kinase 1 (ERK1), is a member of the MAPK family that functions as a central player in the Ras/Raf/MEK/ERK pathway [2,5]. This pathway has been linked to many pathological disorders, with particular emphasis on some oral diseases, inflammatory disorders, and cancer [2,3]. Consequently, MAPK3 is a promising therapeutic target for developing novel small-molecule inhibitors, because it plays a critical role in intracellular signal transduction.

The MAPK/ERK pathway is activated through extracellular growth factors, cytokines, and environmental stress stimuli that trigger sequential phosphorylation events leading to transcriptional regulation of genes involved in cell cycle progression and survival [2,6]. Aberrant activation of MAPK3 leads to uncontrolled cell proliferation, angiogenesis, metastasis, and resistance to apoptosis [2,5]. Thus, the overexpression or constitutive activation of MAPK3 has been documented in several types of cancer, such as breast cancer, lung cancer, colorectal cancer, melanoma, and hepatocellular carcinoma [2,5]. Oral diseases continue to be a significant public health problem, impacting millions of people globally. Chronic inflammatory oral conditions such as periodontitis, oral lichen planus, oral submucous fibrosis, and oral squamous cell carcinoma (OSCC) are characterised by persistent inflammatory signalling, oxidative stress, tissue destruction, and abnormal cellular responses. Several studies have shown that MAPK3-mediated pathways are vital for the progression and pathogenesis of these disorders [3,7].

Oral squamous cell carcinoma (OSCC) is one of the most aggressive and common head and neck malignancies [8,9]. The five-year survival rate of OSCC has remained poor despite the many advances in surgery, chemotherapy, and radiotherapy owing to late diagnosis, local invasion, metastasis, and therapeutic resistance [8,9]. In OSCC, activation of MAPK3 signalling is associated with increased tumor proliferation, EMT, invasion, angiogenesis, and decreased apoptosis [3,10].

Apart from oral cancer, MAPK3 also plays a role in chronic inflammatory oral diseases. Periodontitis is a multifactorial inflammatory disease in which the inflammation of periodontal tissues and bone results in loss of periodontal structure; the inflammatory pathways involving MAPK activation are activated in the gingival fibroblasts, osteoclasts, and immune cells [11]. Persistent ERK signalling is associated with the production of cytokines and degradation of connective tissue and bone [12].

Despite the high number of kinase inhibitors discovered and optimised, the structure of the active site, the high requirement of chemical diversity, and the presence of activity cliffs within a class of structurally related compounds still make optimisation difficult [13]. The traditional method used for drug discovery is time-consuming, labour-intensive, and costly. As such, computational methods like quantitative structure–activity relationship (QSAR) modelling, molecular docking, molecular dynamics (MD) simulation, density functional theory (DFT), and machine learning (ML) virtual screening have become very significant in today’s medicinal chemistry field [14,15].

Hence, the present study involved the development of an integrated computational methodology for the identification and optimisation of potent inhibitors of MAPK3 by applying machine learning-based QSAR modelling and utilising molecular docking, DFT calculation, MD simulation, and MM-GBSA analysis. A set of experimentally validated MAPK3 inhibitors was retrieved from the ChEMBL database and used to build predictive models, using molecular descriptors and Morgan fingerprints. The results of this work could be informative to the development of new therapies beyond oral SCC and other oral inflammatory and proliferative diseases involving dysregulated MAPK signalling.

## 2. Results

### 2.1. Dataset Distribution, Chemical Space, and Applicability Domain Analysis

**Figure 1:**
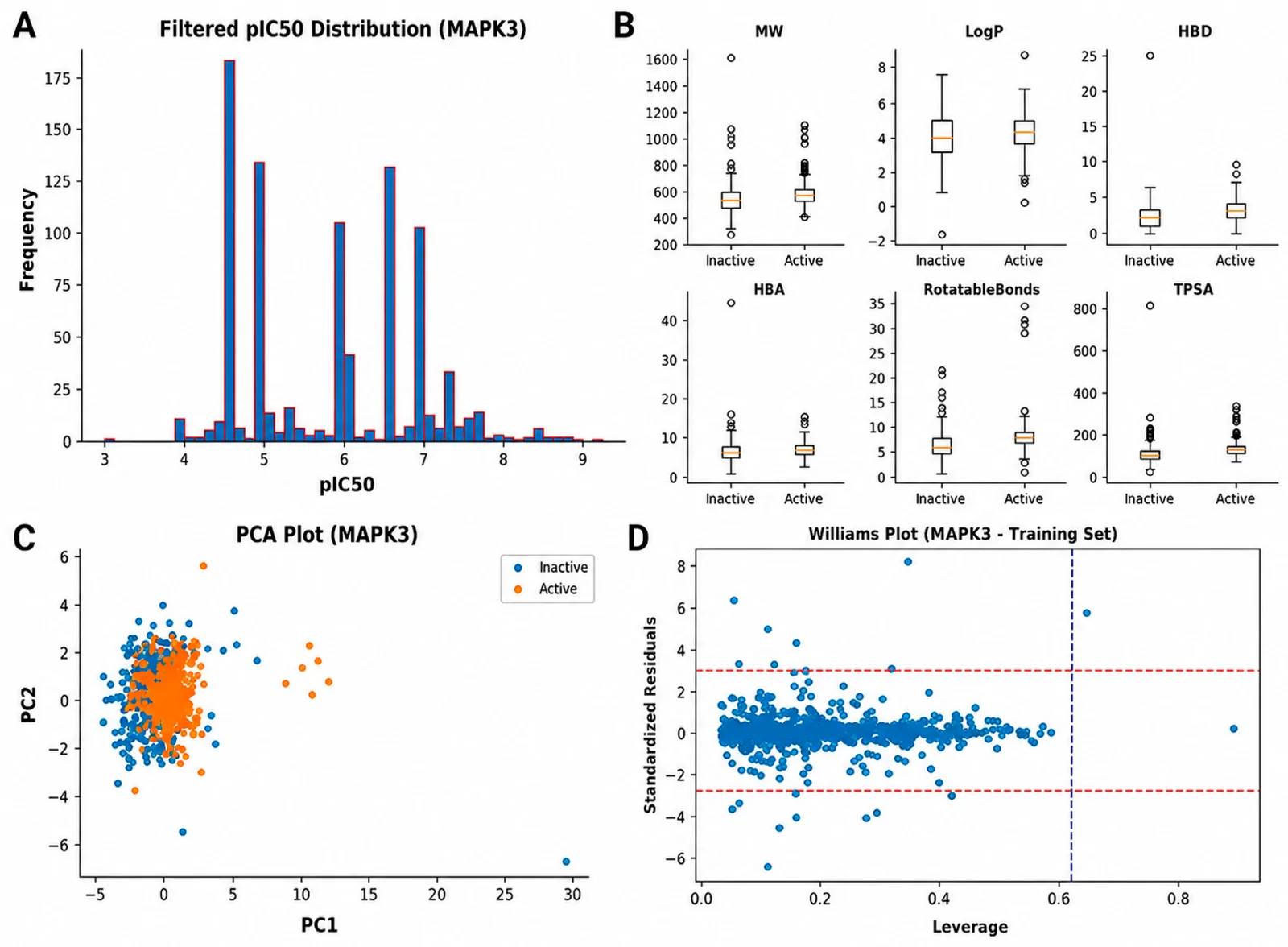
Exploratory data analysis, chemical space distribution, and applicability domain assessment of MAPK3 inhibitors. (**A**) Distribution of *pIC*_50_ values for the curated MAPK3 inhibitor dataset showing activity spread and moderate imbalance across potency ranges. (**B**) Boxplot comparison of key molecular descriptors between active and inactive compounds, including MW, LogP, HBD, HBA, rotatable bonds, and TPSA. (**C**) Principal component analysis (PCA) plot illustrating the chemical space distribution and overlap between active and inactive MAPK3 inhibitors. (**D**) Williams plot showing applicability domain analysis of the final QSAR model, with leverage threshold and standardised residual limits indicating model reliability and influential compounds.

After data preprocessing, activity standardisation, duplicate removal, and outlier filtering, 907 curated MAPK3 inhibitors were retained. The *pIC*_50_ distribution profile (Figure 1A) revealed a wide range of biological activities that spanned from about *pIC*_50_ 3.0 to above 9.0. The histogram showed that the dataset contained moderate activity imbalance, with several peaks of concentration between *pIC*_50_ values 4.5 and 7.0. This imbalance is often seen in kinase inhibitor datasets from various experimental assays and chemical series. Importantly, the dataset also contained a subset of highly potent compounds with *pIC*_50_ > 8.0, which indicated the presence of strong MAPK3 inhibitors that could be used for predictive modelling and hit prioritisation.

The difference between active and inactive compounds was measurable using physicochemical descriptor analysis (Figure 1B). The average molecular weight (MW), LogP, hydrogen bond acceptor (HBA), topological polar surface area (TPSA), and the number of rotatable bonds were found to be slightly higher for active compounds than for inactive compounds. On the other hand, the number of hydrogen bond donors (HBDs) exhibited only small differences between the two classes. A higher molecular size and lipophilicity of the active inhibitors can help in better binding to the ATP-binding pocket and possibly better hydrophobic interactions in the kinase domain. Likewise, the high TPSA and HBA values indicate that polar interactions and hydrogen bond properties play a major role in the binding affinity of MAPK3. The broader descriptor spread observed among active compounds also reflects the structural diversity characteristic of kinase inhibitor scaffolds.

The chemical space distribution for the dataset was visualised using principal component analysis (PCA) with standardised molecular descriptors (Figure 1C). The first two principal components accounted for around 81.1% of the total variance, with the first principal component accounting for 58.86% and the second principal component accounting for 22.28%. The PCA score plot showed a moderate overlap between active and inactive compounds, indicating that compounds with similar global physicochemical properties can have quite different biological activities. A few clusters and outliers were also identified, suggesting moderate scaffold diversity in the dataset. The observed overlap further supports the presence of activity cliffs, where relatively minor structural modifications produce substantial changes in inhibitory potency, a phenomenon commonly encountered in kinase-focused SAR datasets.

The applicability domain (AD) analysis based on the Williams plot showed the robustness and reliability of the final QSAR model (Figure 1D). Most compounds were distributed within the accepted applicability region defined by standardised residuals between ±3 and leverage values below the warning leverage threshold (h* = 0.6248). A small number of compounds were observed to be above these limits, suggesting that there are few influential outliers or molecules with extreme structures. Around 2.48% of compounds were detected as residual outliers, and only 0.28% showed high leverage effects. The model is suitable for virtual screening applications because the low number of influential compounds shows that most of the predictions generated by the model are statistically reliable and are within the range of the compound space defined by the model.

### 2.2. Machine Learning Validation, Feature Importance Analysis, and Hit Prioritisation

**Figure 2:**
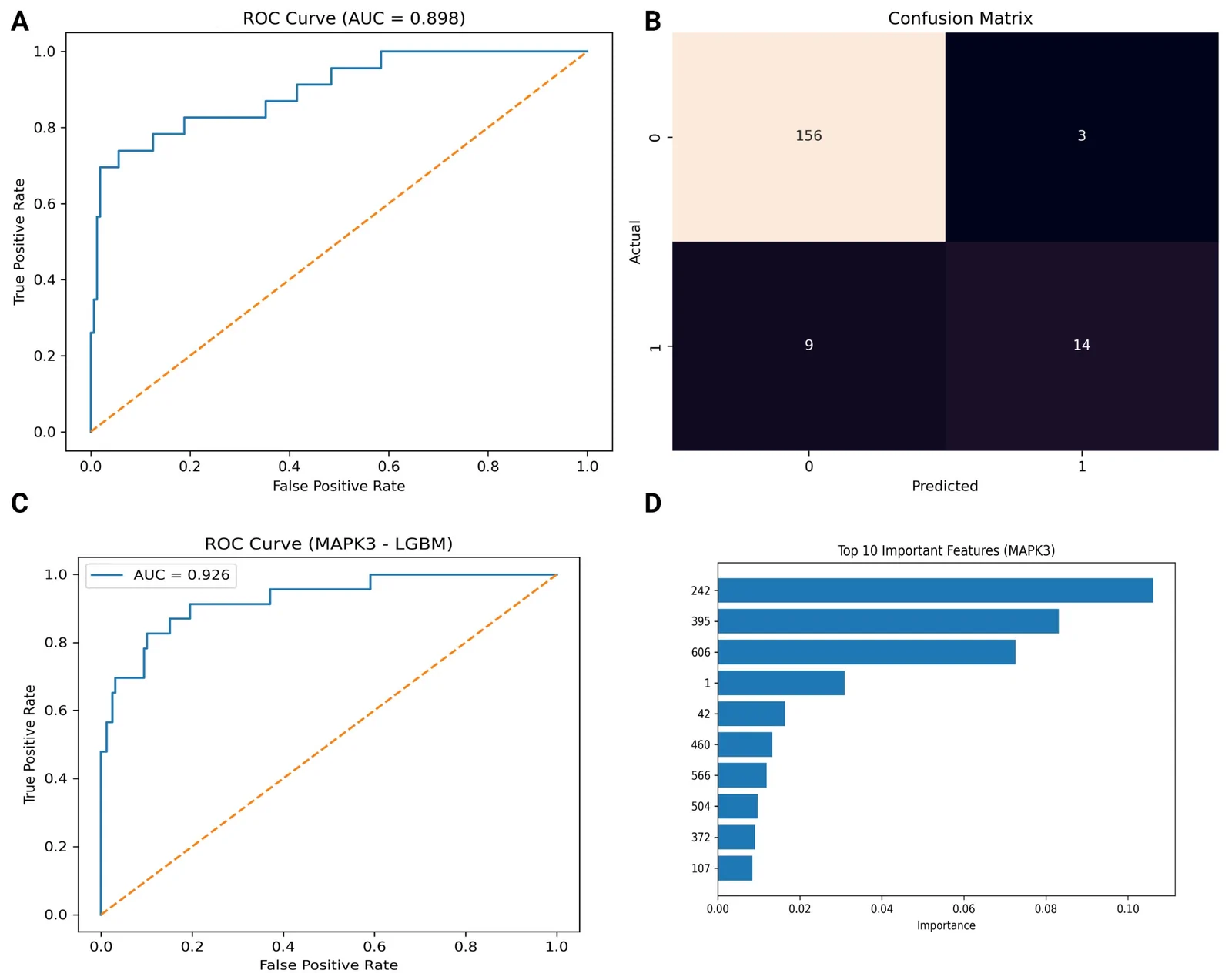
Machine learning validation, feature importance analysis, and prioritisation of potent MAPK3 inhibitors using the LightGBM QSAR model. (**A**) Receiver operating characteristic (ROC) curve demonstrating classification performance of the final model with an AUC of 0.898. (**B**) Confusion matrix showing classification outcomes for active and inactive compounds in the external test set. (**C**) ROC curve of the optimised LightGBM classifier demonstrating improved predictive discrimination with an AUC of 0.926. (**D**) Top 10 permutation-important features contributing to MAPK3 inhibitory activity prediction, highlighting the importance of fingerprint-derived structural descriptors. The final LightGBM model prioritised the top 20 potent MAPK3 inhibitors (Supplementary File S1), which were subsequently selected for molecular docking and further structure-based computational analyses.

The Light Gradient Boosting Machine (LightGBM) model was the most promising machine learning system in terms of predictive performance and was chosen as the final MAPK3 QSAR model. The classification-oriented validation proved to have very good discriminative power between active and inactive compounds. The receiver operating characteristic (ROC) curve was used to evaluate the classification accuracy, achieving an area under the curve (AUC) of 0.898 (Figure 2A), which demonstrated good classification accuracy and effective separation of the compounds based on the inhibitory activity. (Figure 2A corresponds to the initial LightGBM model evaluated under internal scaffold-based cross-validation settings.) Despite the strict external validation approach based on scaffolds, the ROC curve was still significantly better than the random classification level.

Importantly, the cross-validation R^2^ value (≈0.067) refers to the regression-based QSAR formulation applied under strict scaffold splitting conditions, where prediction of continuous activity values is inherently challenging due to chemical diversity and limited activity distribution. In contrast, ROC-AUC evaluates the classification performance of the LightGBM model in distinguishing active versus inactive compounds; therefore, these metrics reflect different modelling objectives and are not directly comparable.

To improve the performance of the optimised LightGBM classifier, another ROC analysis was performed specifically for this model, which resulted in an AUC value of 0.926 (Figure 2C), again confirming its strong predictive power and stability of the final machine learning structure. (Figure 2C represents the hyperparameter-optimised LightGBM model evaluated after model tuning on the final validation configuration, leading to improved classification performance compared with the baseline model in Figure 2A.) The steep early increase in the ROC curve at low false positive rates further demonstrates the ability of the model to prioritise highly potent inhibitors during virtual screening procedures. The findings validate the applicability of the LightGBM model for discovering new MAPK3 inhibitors from diverse compound libraries.

Several fingerprint-derived variables were found to be the most relevant for the model performance using the permutation feature importance analysis (Figure 2D). The dominant features were mainly related to Morgan fingerprint bits instead of global physicochemical descriptors, suggesting that local atomic environments and specific substructural motifs are important for the inhibitory activity of MAPK3. A relatively low number of features were responsible for a high proportion of prediction accuracy, indicating the presence of strong structure–activity relationships (SARs) within the dataset. This behaviour is in line with the datasets of the kinases: slight changes in the scaffold can lead to significant shifts in potency. The moderate variance observed during scaffold-based cross-validation may be in part attributed to the uneven distribution of feature importance, a feature characteristic that facilitates the occurrence of activity cliffs.

After model optimisation and validation, the optimised LightGBM model was employed to rank the most important MAPK3 inhibitors according to their predicted activity. The top 20 compounds identified by the model were listed in Supplementary File S1 and then chosen for molecular docking and further structure-based analysis. The *pIC*_50_ values of these compounds were experimentally verified and ranged from about 7.3 to 8.9, which represents strong inhibitory potency against MAPK3. Several prioritised compounds, including CHEMBL4434915, CHEMBL3544964, CHEMBL4650280, CHEMBL583042, and CHEMBL3409588, demonstrated favourable physicochemical characteristics associated with kinase inhibition, including moderate molecular weight, balanced lipophilicity, acceptable polar surface area, and suitable hydrogen-bonding capacity. Perhaps most importantly, a common feature of almost all of the top-ranked compounds was the presence of heterocyclic kinase-binding scaffolds alongside halogen-substituted aromatic residues, both of which are known to increase the selectivity and binding affinity of kinase inhibitors. These highly potent compounds were identified, showcasing the utility of the integrated QSAR–machine learning workflow for prioritisation of promising MAPK3 inhibitor candidates for subsequent molecular docking, molecular dynamics simulation, MM-GBSA, and DFT studies.

### 2.3. Molecular Docking and ADMET Analysis

**Figure 3:**
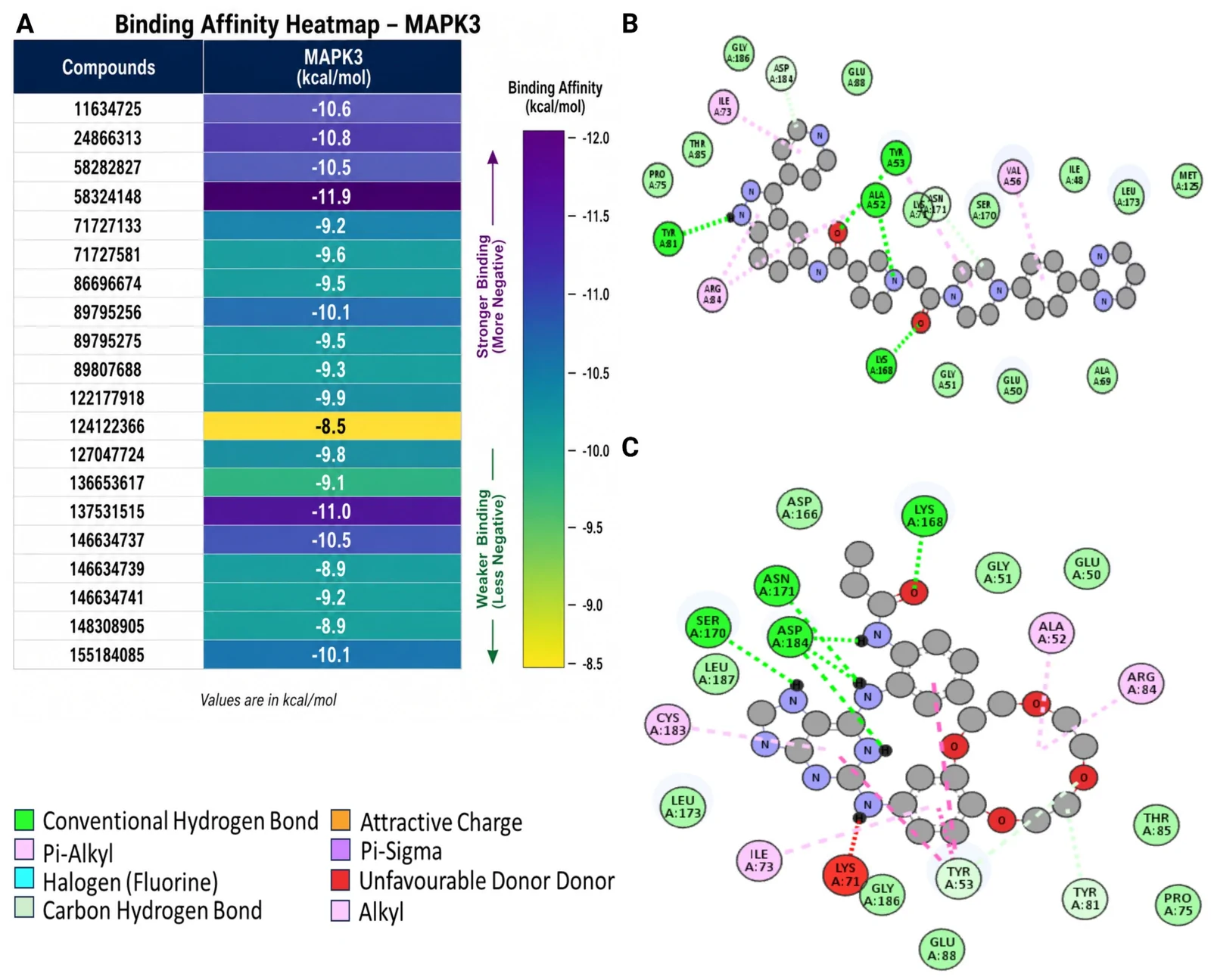
Molecular docking analysis of prioritised MAPK3 inhibitors. (**A**) Heatmap showing docking binding affinities of the top-ranked MAPK3 inhibitors against the MAPK3 protein target, expressed in kcal/mol. More negative values indicate stronger binding affinity. (**B**) Two-dimensional interaction diagram of compound 58324148 within the MAPK3 active site showing hydrogen bonding, hydrophobic, π–alkyl, π–sigma, and electrostatic interactions with key amino acid residues. (**C**) Two-dimensional interaction diagram of compound 137531515 within the MAPK3 binding pocket illustrating stabilising hydrogen bonding and hydrophobic interactions with catalytic residues.

The highest-ranked machine learning prioritised MAPK3 inhibitors were then analysed using molecular docking to explore their binding affinity and interaction with the MAPK3 active site. The binding affinities of several compounds obtained from the docking results were found to be very good, and the calculated binding energies of these compounds ranged from about −8.5 to −11.9 kcal/mol (Figure 3A). Among the screened compounds, compound 58324148 exhibited the strongest binding affinity with a docking score of −11.9 kcal/mol, followed by compounds 137531515 (−11.0 kcal/mol), 24866313 (−10.8 kcal/mol), and 11634725 (−10.6 kcal/mol). The predominance of highly negative docking energies indicates favourable ligand accommodation within the MAPK3 catalytic pocket and suggests strong thermodynamic stability of the receptor–ligand complexes.

The heatmap distribution also indicated that the majority of prioritised compounds showed consistently high binding energies below −9.0 kcal/mol. A few compounds showed relatively low binding affinities, but their score was still in the acceptable range typical of biologically active kinase inhibitors. The results confirm the predictiveness of the QSAR–machine learning workflow to find compounds with high MAPK3 inhibitory capacity. Furthermore, comparison with reference MAPK3 inhibitors showed that Ulixertinib (−10.0 kcal/mol), Ravoxertinib (−9.6 kcal/mol), and the co-crystallised ligand (−10.7 kcal/mol) exhibited lower or comparable binding affinities than the top-ranked novel compounds (Figure S2), indicating improved docking performance of compounds 58324148 and 137531515.

Detailed interaction analysis of compound 58324148 (Figure 3B), which showed the highest docking score, revealed multiple stabilising interactions within the MAPK3 binding pocket. The ligand established conventional hydrogen bonds with key residues, such as Lys54 and Ala52, which are essential residues in the ATP-binding site of MAPK3. The complex was further stabilised by additional hydrophobic interactions, which involved π–alkyl and alkyl interactions with residues like Lys71, Ala52, and Val56. Other interactions, such as carbon–hydrogen bonding and π–sigma interactions, were also found, which helped to improve the binding stability. The aromatic heterocyclic scaffold and halogen-substituted phenyl groups of the ligand appeared to facilitate efficient occupancy of the kinase hydrophobic pocket, thereby enhancing binding affinity.

Similarly, compound 137531515 (Figure 3C) demonstrated strong binding interactions with MAPK3, consistent with its high docking score of −11.0 kcal/mol. The ligand formed several hydrogen bonds with the residues Asp104, Lys54, Ser170, and Asn171, which indicates its stable bonding within the active site. A series of hydrophobic and electrostatic interactions, including π–alkyl and attractive charge interactions, was also observed with residues like Ile73, Tyr53, and Arg84. Based on the interaction profile, both polar and hydrophobic contacts appear to play a synergistic role in the stabilisation of the ligand within the kinase domain. Importantly, both compounds had similar catalytic regions that are linked to ATP binding, which is consistent with their proposed mechanism as competitive kinase inhibitors.

In comparison, the co-crystallised ligand primarily retained interactions with core hinge region residues but exhibited fewer hydrophobic and electrostatic contacts, while Ulixertinib and Ravoxertinib showed interaction patterns dominated by limited hydrogen bonding and weaker residue engagement, particularly with key residues, such as Lys54, Ala52, and Asp104, which were more extensively engaged by the novel compounds. This suggests that the newly identified compounds achieve a more complete occupancy of the MAPK3 binding pocket, contributing to their higher docking scores.

Overall, the molecular docking results demonstrated that the prioritised compounds possess favourable binding characteristics against MAPK3, with strong interaction networks involving critical catalytic residues. The docking analysis also confirmed the machine learning predictions and suggested compounds 58324148 and 137531515 as computationally prioritised MAPK3 binding candidates for subsequent density functional theory (DFT), molecular dynamics simulation (MDS), and molecular mechanics–GBSA (MM-GBSA) analysis, with improved binding performance compared to co-crystallised and known MAPK3 inhibitors.

The ADMET radar profiles indicate that the co-crystal and both compounds (58324148 and 137351515) generally fall within an acceptable drug-likeness space, with the co-crystal showing the most balanced overall physicochemical properties across parameters such as molecular weight, lipophilicity, polarity (TPSA), and solubility-related descriptors (Figure S3). The reference compounds (Ulixertinib and Ravoxertinib) display comparatively higher deviations in certain descriptors, reflecting less uniform drug-likeness balance.

In the toxicity assessment, the control compounds exhibit distinct endpoint-specific liabilities, with Ulixertinib showing relatively higher mutagenicity signals and Ravoxertinib showing stronger cardiotoxicity-related risk. The co-crystal demonstrates comparatively lower predicted toxicity across most endpoints, suggesting a more favourable safety baseline. Among the compounds, 58324148 and 137351515 show moderate and variable toxicity signals, particularly in hepatotoxicity and carcinogenicity predictions, but without consistent high risk across all evaluated endpoints. Overall, the integrated ADMET–toxicity comparison suggests that the co-crystal and at least one of the novel scaffolds retain competitive drug-like and safety profiles relative to established controls, supporting their progression for further optimisation and validation.

### 2.4. DFT and Frontier Molecular Orbital Analysis

The electronic properties, chemical reactivity, and charge distribution characteristics of the two most promising compounds obtained by molecular docking (137531515 and 58324148) were calculated by density functional theory (DFT). To understand the importance of stability and electron transfer capability of the compound, the Frontier molecular orbital (FMO) analysis was performed to study the highest occupied molecular orbital (HOMO) and lowest unoccupied molecular orbital (LUMO) and the energy gap between them. Furthermore, these quantum descriptors were used to interpret ligand–MAPK3 interactions by linking HOMO regions with potential electron donation toward electrophilic amino acid residues in the active site, while LUMO regions indicate sites capable of accepting electron density during charge-transfer-driven stabilisation of the receptor–ligand complex.

**Figure 4:**
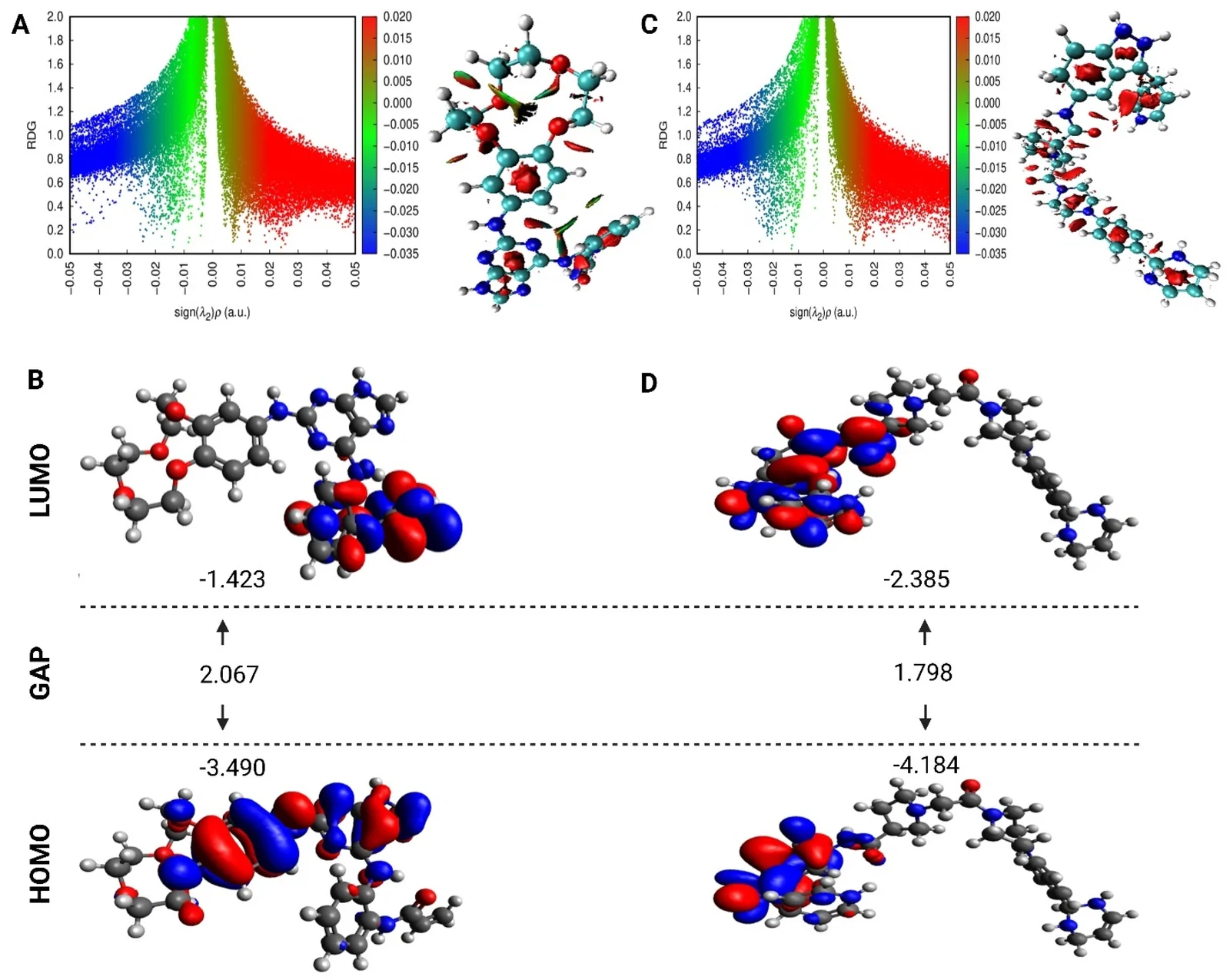
Density functional theory (DFT) and frontier molecular orbital analysis of selected MAPK3 inhibitors. (**A**) Reduced density gradient (RDG) analysis and non-covalent interaction visualisation of compound 137531515. (**B**) HOMO–LUMO orbital distribution and energy gap analysis of compound 137531515 showing a HOMO–LUMO gap of 2.067 eV. (**C**) Reduced density gradient (RDG) analysis and non-covalent interaction visualisation of compound 58324148. (**D**) HOMO–LUMO orbital distribution and energy gap analysis of compound 58324148 showing a HOMO–LUMO gap of 1.798 eV. Panels (**A**,**C**): red = steric repulsion, blue = attractive non-covalent interactions. Panels (**B**,**D**): red and blue simply represent opposite orbital phases of the HOMO and LUMO wavefunctions.

The compound 137531515 (Figure 4A,B) has an energy gap of 2.067 eV with an HOMO energy level of −3.490 eV and a LUMO energy level of −1.423 eV. The moderate HOMO–LUMO gap indicates high electronic flexibility and favourable chemical reactivity, which could enable efficient interaction with the active site of MAPK3. The HOMO electron density was localised mainly on the aromatic heterocyclic framework of the molecule, suggesting that these regions can serve as electron donation centres when interacting with the receptor. By comparison, the LUMO density was primarily concentrated around the electron-deficient regions containing the heteroatoms, indicating possible intermolecular charge transfer interactions and electron acceptance.

Specifically, electron-rich aromatic and heterocyclic rings are identified as major HOMO-dominated regions contributing to π-donation, whereas heteroatom-containing functional groups (such as nitrogen- and oxygen-bearing moieties) correspond to LUMO-localised regions that facilitate electrophilic interactions and stabilise binding within the MAPK3 pocket through charge-transfer effects.

The reduced density gradient (RDG) analysis of 137531515 also confirmed the presence of several weak non-covalent interactions, such as van der Waals and steric effects. The distribution of green isosurfaces in the RDG map showed stabilising weak interactions, and the red regions showed steric repulsions. These results suggest that the compound can form the necessary intermolecular interactions in the MAPK3 binding pocket.

Compound 58324148 (Figure 4C,D) exhibited a HOMO energy of −4.184 eV and a LUMO energy of −2.385 eV, resulting in a smaller HOMO–LUMO energy gap of 1.798 eV compared with compound 137531515. The HOMO–LUMO energy gap of 1.798 eV (vs. 2.067 eV for 137531515) indicates higher chemical softness and enhanced electronic reactivity, supporting stronger charge-transfer interactions with MAPK3 binding residues. The HOMO distribution was localised around the conjugated aromatic and heterocyclic scaffold, while the LUMO distribution was localised near electron-withdrawing functional groups and heteroatoms that are involved in receptor interactions. In this compound, conjugated aromatic scaffolds act as principal HOMO-dominant electron-donating regions, while electron-withdrawing substituents (including heteroatoms, such as N and O) define LUMO-dominant sites that promote electrophilic stabilisation and strengthen intermolecular contacts within the MAPK3 active pocket.

The RDG analysis of compound 58324148 also revealed large areas of non-covalent interactions, with significant green isosurfaces indicating strong van der Waals and dispersion interactions. The increased density of stabilising interaction regions compared with compound 137531515 may partially explain the superior docking affinity exhibited by compound 58324148. The ELF and frontier orbital analyses both indicate that compound 58324148 has an excellent electronic structure for intermolecular interactions and kinase binding.

Overall, the DFT results showed that both compounds have quantitatively distinct electronic characteristics, as evidenced by HOMO–LUMO gaps of 2.067 eV (137531515) and 1.798 eV (58324148), which are suitable for strong kinase inhibitory potential. The relatively small HOMO–LUMO gaps, favourable charge distribution patterns, and extensive weak interaction regions support the stability of the receptor–ligand complexes observed during molecular docking. Among both compounds, 58324148 displayed slightly higher electronic reactivity and stronger non-covalent interaction potential, correlating with its superior docking affinity against MAPK3.

### 2.5. Molecular Dynamics Simulation Analysis

To further investigate the dynamic stability and structural properties of the selected MAPK3 inhibitor complexes, both 58324148 and 137531515 bound to MAPK3 were subjected to 200 ns molecular dynamics (MD) simulations. To evaluate the conformational stability of the receptor–ligand complexes, several trajectory-based parameters were analysed, such as root mean square deviation (RMSD), root mean square fluctuation (RMSF), and hydrogen bond occupancy.

**Figure 5:**
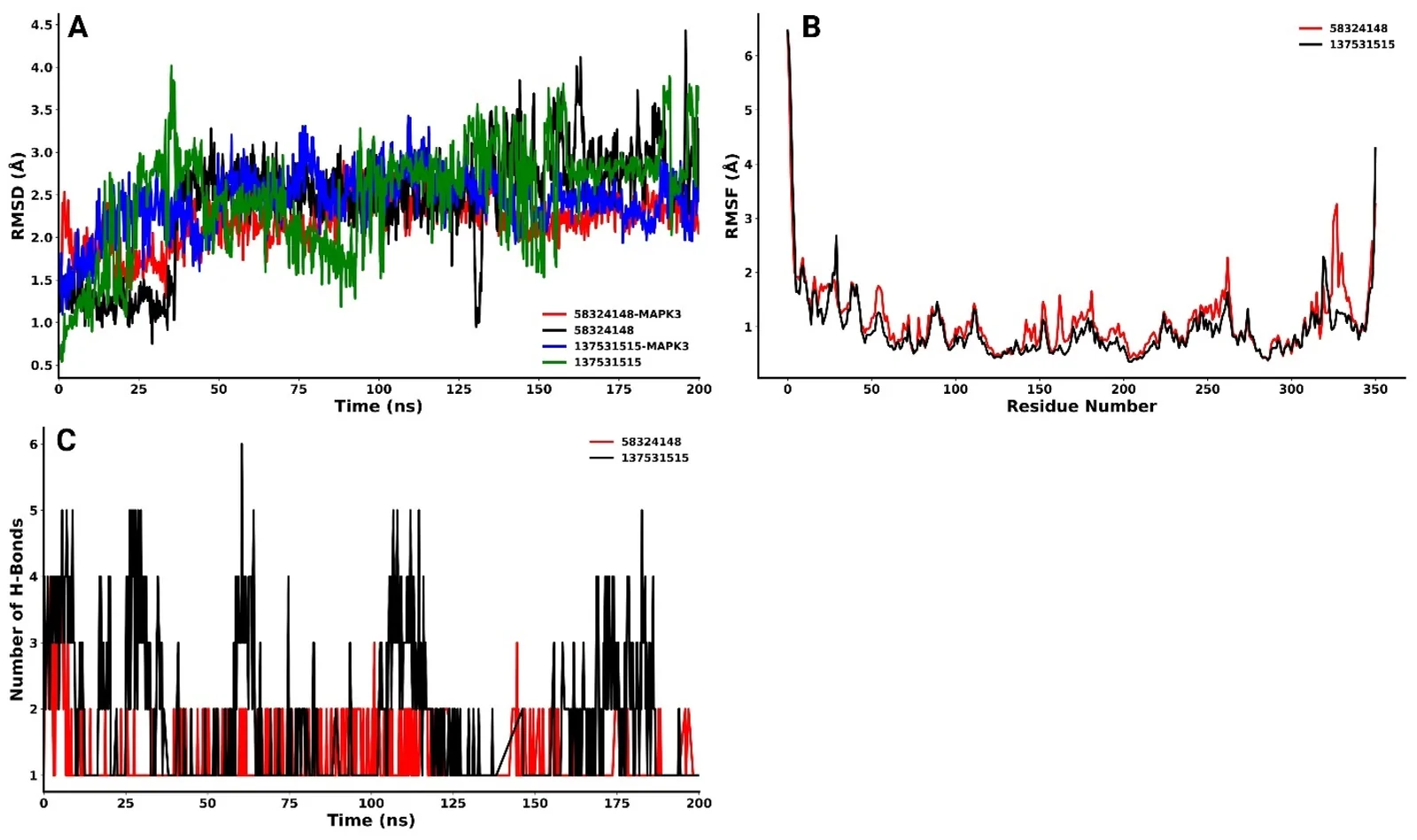
Molecular dynamics simulation analysis of MAPK3 complexes with compounds 58324148 and 137531515. (**A**) Root mean square deviation (RMSD) trajectories of MAPK3 backbone atoms and ligand conformations during the 200 ns simulation period. (**B**) Root mean square fluctuation (RMSF) profiles showing residue-level flexibility of MAPK3 in complex with compounds 58324148 and 137531515. (**C**) Hydrogen bond occupancy analysis showing the number of intermolecular hydrogen bonds formed between MAPK3 and the ligands throughout the simulation.

The RMSD fluctuation of both the ligand-bound systems showed good stability during the simulation time (Figure 5A). During the initial period of the simulation, the MAPK3–58324148 complex achieved equilibrium but subsequently fluctuated around its initial state, with RMSD values mostly in the range of 2.0 to 2.8 Å. Conversely, the complex MAPK3–137531515 had slightly higher conformational deviations throughout the later stages of the simulation, with RMSDs greater than 3.0 Å at certain points. In both complexes, the overall structure remained intact despite these changes, with no evidence of significant destabilisation or dissociation of the ligands from the MAPK3 binding pocket, suggesting that the ligands in both complexes were well accommodated. For comparative evaluation, MD simulations of the co-crystal MAPK3 complex and known inhibitors Ulixertinib and Ravoxertinib were also analysed (Figure S4). The co-crystal system exhibited relatively stable RMSD behaviour, whereas both reference inhibitors showed slightly higher fluctuations compared to 58324148, indicating comparatively reduced structural stability.

The flexibility of the protein residues was also explored using RMSF analysis (Figure 5B). The backbone motion for most of the amino acid residues, as reflected by the fluctuation values, was relatively low, with values less than 2.0 Å indicating stable backbone dynamics overall for the kinase structure. The majority of higher fluctuations were found in terminal loop regions and flexible solvent-exposed segments, characteristic of kinase proteins. The MAPK3–137531515 complex showed slightly higher RMSF in some regions of the residues compared to the MAPK3–58324148 complex, suggesting a slightly higher degree of flexibility in the selected regions due to the binding of the ligand. However, the residues involved in the ligand interaction in the ATP-binding region remained relatively stable during the simulation. In control systems, RMSF profiles of the co-crystal and reference inhibitors (Ulixertinib and Ravoxertinib) showed comparable but slightly elevated fluctuations in loop regions, while catalytic site residues remained conserved across all systems, indicating preservation of structural integrity within the MAPK3 active site.

During the simulation time, intermolecular interactions between the ligands and MAPK3 remained strong and were identified and characterised using hydrogen bond occupancy analysis (Figure 5C). The compound 58324148 formed around one to three hydrogen bonds that remained stable over the full trajectory, indicating stable receptor engagement. In contrast, compound 137531515 exhibited greater variation in hydrogen bond numbers, occasionally forming up to five or six hydrogen bonds during specific intervals. The changing hydrogen-bonding patterns of these two systems suggest the existence of adaptive interaction behaviour in the kinase binding site while maintaining the stability of the overall complex. The control inhibitors showed comparatively lower and less persistent hydrogen bond occupancy relative to 58324148, whereas the co-crystal complex maintained stable but fewer hydrogen bond interactions throughout the simulation, further supporting stronger ligand engagement by 58324148.

Collectively, the MD simulation results demonstrated that both compounds formed structurally stable complexes with MAPK3 over the 200 ns simulation period. The structural fluctuations and RMSD values of compound 58324148 were comparatively lower and more stable than those of compound 137531515, indicating better conformational stability. The results are also consistent with the docking and DFT results and suggest that compound 58324148 is a good candidate for an inhibitor of MAPK3.

### 2.6. Dynamic Correlation, Conformational Stability, and MM-GBSA Energy Analysis

After MD simulation, dynamic cross-correlation matrix (DCCM), free energy landscape (FEL), radius of gyration (Rg), and MM-GBSA analyses were performed to further characterise the dynamic behaviour and energetic stability of the MAPK3–ligand complexes, respectively.

The DCCM analysis of the MAPK3–58324148 complex (Supplementary File S2 Figure S1A) revealed substantial positively correlated motions across several residue regions within the kinase structure, represented by red correlation zones. These correlated movements suggest coordinated domain motions that may contribute to the stabilisation of the ligand-bound conformation. The anti-correlated motions were observed in limited regions (blue), which means that the dynamic communication is relatively stable throughout the protein. Likewise, there were correlated motions between residues in the MAPK3–137531515 complex (Figure S1D), but there was a relatively larger amount of anti-correlated motions in some regions, indicating slightly more dynamic flexibility than the 58324148 complex. In comparison, the co-crystal system and reference inhibitors (Ulixertinib and Ravoxertinib) exhibited intermediate correlation patterns (Figure S5), with more distributed anti-correlated regions than 58324148 but less than 137531515, supporting the intermediate stability trend observed in RMSD and RMSF analyses.

The simulated systems were analysed by free energy landscape (FEL) analysis, which gave insights into the conformational stability of the systems. In the case of the MAPK3–58324148 complex (Figure S1B), a low-energy basin was identified with numerous minima, suggesting that the system had a stable conformational state along the course of the simulation time. The complex MAPK3–137531515 (Figure S1E) exhibited a wider and more spread-out energy landscape, with multiple local minima, indicating a more variable and flexible arrangement throughout the simulation. The energy basin of the compound 58324148 is more compact, explaining its higher conformational stability in the MAPK3 binding pocket. The control systems (co-crystal, Ulixertinib, and Ravoxertinib) showed intermediate FEL profiles, with co-crystal displaying a more compact energy basin compared to both reference inhibitors, but still less restricted than 58324148, suggesting the highest conformational confinement for 58324148 among all systems.

The compactness of the protein–ligand complexes was analysed by radius of gyration (Rg). The Rg value of the MAPK3–58324148 complex (Figure S1C) remained relatively constant throughout the simulation, ranging from about 21.7 to 22.3 Å, which suggests that the protein complexes remain compact and structurally intact. The MAPK3–137531515 complex also showed stable Rg behaviour with slightly larger fluctuation patterns along the trajectory (Figure S1F). No significant Rg deviations were found in both systems, which confirms there were no significant changes in the structure of MAPK3 upon ligand binding. The reference systems showed comparable Rg stability; however, Ulixertinib and Ravoxertinib exhibited slightly broader fluctuation ranges than 58324148, indicating marginally reduced compactness in comparison.

The energetic favourability of both complexes was also confirmed by the MM-GBSA energy decomposition analysis (Figure S1G,H). The binding free energy contribution of the MAPK3–58324148 complex was found to be significantly more favourable across the whole simulation trajectory, with the van der Waals contribution being the major contributor. Coulombic interactions and lipophilic interactions also played positive roles in complex stability, and the solvation-related energy components were found to play partial negative roles, which is the expected binding nature in kinase–ligand systems. Importantly, the overall binding energy profile for MM-GBSA was relatively stable during the simulation, indicating good stability of the ligand within the binding pocket.

Likewise, the energy profile for MM-GBSA showed favourable results for the MAPK3–137531515 complex (Figure S1H), with slightly larger fluctuations in the total binding energy when compared with the 58324148 system. Ligand binding was stabilised by van der Waals interactions and also by electrostatic and hydrogen-bonding interactions. The somewhat larger energetic variability for the 137531515 complex is consistent with the increased conformational flexibility revealed in RMSD, DCCM, and FEL analyses. The control inhibitors (Ulixertinib and Ravoxertinib) showed comparatively weaker MM-GBSA binding free energies than 58324148 (Figure S6), with higher fluctuation ranges, while the co-crystal system demonstrated stable but intermediate binding energy profiles, further supporting the superior energetic stability of 58324148 across all evaluated systems.

Overall, the combined MD simulation and MM-GBSA results demonstrated that both compounds formed stable and energetically favourable complexes with MAPK3 throughout the 200 ns simulation period. It was observed that 58324148 had consistently better structural stability, conformational stability, lower conformational fluctuations, more compact low-energy conformational states, and stronger energetic favourability. The results indicate that compound 58324148 is a promising lead compound for further optimisation and experimental testing of MAPK3 inhibitors.

## 3. Discussion

MAPK3 (ERK1) is an important regulator of the Ras/Raf/MEK/ERK signalling pathway and is involved in many inflammatory and proliferative oral diseases, such as oral squamous cell carcinoma (OSCC), periodontal disease, oral lichen planus, and oral submucous fibrosis [16,17]. An abnormal activation of MAPK3 leads to excess proliferation, inflammation, angiogenesis, and apoptosis resistance, making it a potential target for therapeutic intervention [18,19]. An integrated AI-powered computational workflow that incorporates QSAR modelling, machine learning, molecular docking, DFT calculations, molecular dynamics (MD) simulation, and MM-GBSA analysis was used in the present study to discover potent inhibitors of MAPK3 [20,21,22].

The curated dataset exhibited a wide structural and biological diversity, typical for kinase inhibitor datasets [23,24]. The active compounds typically had higher molecular weight, lipophilicity, polar surface area, and hydrogen-bonding ability than the inactive compounds, indicating that these are important features for strong binding to the kinases [25,26]. The moderate overlap of active and inactive compounds in the PCA indicated the existence of activity cliffs, in which only small changes in structure would lead to significant differences in potency [27,28]. These types of activities are prevalent in kinase inhibitor datasets and are probably responsible for the relatively low variance observed in the scaffold-based validation [29].

Overall, LightGBM showed the highest predictive performance among all the machine learning models evaluated, with a very high ROC-AUC value, which means it had very good discrimination between active and inactive compounds [30]. The model was also scaffold-based and validated, and it maintained good predictive ability for structurally different compounds [31]. The additional importance of local substructural motifs in MAPK3 inhibition was further indicated by permutation importance analysis, which showed that fingerprint-derived structural descriptors were more important than the global physicochemical descriptors [32,33].

The machine learning predictions were further confirmed by molecular docking analysis, with the top-ranked compounds showing excellent binding affinities to MAPK3. The compounds 58324148 and 137531515 showed the best docking scores and made several stabilising interactions with important active-site residues like Lys54, Ala52, Asp104, Ser170, and Asn171. These interactions included hydrogen bonding, hydrophobic contacts, and electrostatic interactions that helped to ensure the stability of the ligand in the ATP-binding pocket [34,35].

The DFT calculations showed that both compounds exhibited good electronic characteristics, such as a relatively small HOMO–LUMO gap, with good chemical reactivity and intermolecular interaction potential [36,37]. The compound 58324148 exhibited the lowest energy gap and thus has higher electronic softness and stronger binding ability [38]. MD simulation also indicated that both receptor–ligand complexes were stable during the 200 ns simulation time. The RMSD fluctuations, conformational variability, and energetic behaviour of compound 58324148 were found to be more stable and less fluctuating as compared to compound 137531515. MM-GBSA analysis also revealed favourable binding energies dominated primarily by van der Waals interactions [39,40].

Although these are encouraging results, there is a lack of experimental validation and kinase selectivity profiling. Thus, further in vitro and in vivo investigations are required to validate the biological activity, selectivity, and therapeutic potential of the identified compounds. In sum, our current study highlights the potential of using artificial intelligence and structure-based computational methods to expedite the discovery of MAPK3 inhibitors for oral inflammatory and proliferative diseases.

## 4. Materials and Methods

### 4.1. Data Collection and Curation

The *Python* 3.12 (64 bit) ChEMBL 37 web resource client was used to retrieve the bioactivity data for the mitogen-activated protein kinase 3 target (MAPK3; ChEMBL ID: CHEMBL3385) from the ChEMBL database [41,42]. Compounds with experimentally determined inhibitory activity values against MAPK3 were retained. Downstream processing extracted canonical SMILES, ChEMBL compound identifiers, activity values, and units. Data preprocessing was performed to ensure chemical and biological consistency. The canonical SMILES or activity measurements of compounds were filtered out. Only data reported in nanomolars (nM) was included to ensure standardisation of experimental measurements. To minimise assay redundancy and experimental noise, compounds having multiple canonical SMILES were aggregated by taking the average of their *pIC*_50_ values [43].

Biological activities were transformed from *IC*_50_ values to *pIC*_50_ using Equation (1):

(1) $$\rho {IC}_{50\;=\;-log10\left(IC50\times 10^{-9}\right)}$$

Compounds with *pIC*_50_ values less than 3 were excluded as low-activity outliers for a more robust model and to minimise noise from weakly active and inactive compounds. A final curated dataset of 907 unique MAPK3 inhibitors was obtained.

### 4.2. Exploratory Data and Molecular Descriptor Calculation

The distribution of *pIC*_50_ values were analysed using *Matplotlib v 3.11.0* and *Seaborn v 0.13.2* [44] histograms and kernel density estimation plots [42] histograms and kernel density estimation plots. Descriptive statistical parameters were used for determining data spread and balance: mean, standard deviation, quartiles, and minimum and maximum values. A gap analysis was carried out through 50-bin histogram segmentation to find underrepresented activity regions. The number of bins with the most occurrences was compared to the average number of occurrences in each bin to detect distribution imbalance and peaks. This analysis revealed moderate activity imbalance in the dataset, which is typical for kinase inhibitor datasets from different experiments. Canonical SMILES strings were used to calculate physicochemical descriptors with the RDKit toolkit 2026.03.4. Six medicinal chemistry descriptors were developed: molecular weight (MW), octanol/water partition coefficient (LogP), hydrogen bond donors (HBDs), hydrogen bond acceptors (HBAs), topological polar surface area (TPSA), and number of rotatable bonds. The descriptors were selected as they are known to be important in the optimisation of kinase inhibitors, membrane permeability, and binding affinity for the target [45,46]. A *pIC*_50_ threshold of 6.0 was used to classify compounds as active or inactive classes. The Mann–Whitney U test was used for the statistical comparisons between the two classes due to the non-Gaussian distribution of the descriptor data [47]. Statistical significance was defined at *p* < 0.05.

### 4.3. Principal Component, Molecular Fingerprint Generation, and Dataset Preparation

Principal component analysis (PCA) was applied to visualise the chemical space distribution and analyse the clustering patterns among MAPK3 inhibitors [48]. Z-score normalisation was used to normalise the values of the descriptors before applying the PCA transformation. The first two principal components accounted for about 81.1% of the variance: PC1 58.86% and PC2 22.28%. The PCA plot showed moderate chemical diversity with moderate overlapping between active and inactive compounds, suggesting the presence of structurally related compounds with different biological activities. Extended-connectivity Morgan fingerprints were generated using the RDKit toolkit with the following parameters: radius = 2 and bit vector size = 1024 bits [33]. Morgan fingerprints represent circular atom environments and are well-suited for modelling structure–activity relationships (SARs) for kinase inhibitors because they uniquely capture substructural features that are local to the binding sites [33,49]. The fingerprint vectors were then combined with molecular descriptors calculated to form a final feature matrix for machine learning. To develop the models, only numeric columns were saved. Identifiers other than numeric, such as compound names and SMILES strings, were not included in model building. Missing values were checked, and no missing values were detected in either the feature matrix or target vector. The final dataset consisted of: 907 compounds, 1030 predictive features, and 1 target variable (*pIC*_50_).

### 4.4. Scaffold-Based Data Splitting, Feature Selection, and Machine Learning Model Development

Data splitting was carried out on the basis of the structural similarity of the analogues based on the Bemis–Murcko scaffold decomposition performed in RDKit, so as to avoid data leakage and to provide realistic model generalisation [50]. Unique scaffolds were identified for each molecule and separated into training and test sets while ensuring no scaffold overlap between the two subsets. The final split resulted in a training set of 725 compounds, a test set of 182 compounds, and no common scaffolds. This scaffold-based splitting strategy provides a much more rigorous test than random splitting and is more representative of future virtual screening conditions [29]. To prevent test set data leakage, feature selection was performed only on the training set. Low-information and redundant variables were eliminated by variance threshold filtering and feature importance analysis [51]. Initially, 1030 features were present. The selection process resulted in 712 informative features being retained for further model development. Optimisation experiments further reduced the feature space to 150 high-value features. Selected feature matrices were saved separately for reproducibility and further analysis. Selected features were used to evaluate multiple regression algorithms, namely, Random Forest (RF), Extra Trees (ETs), Gradient Boosting (GB), Decision Tree (DT), Linear Regression (LR), k-Nearest Neighbours (KNNs), Support Vector Regression (SVR), Multi-layer Perceptron (MLP), Extreme Gradient Boosting (XGBoost), and Light Gradient Boosting Machine (LightGBM). All the models were built using the scikit-learn library 1.9.0 [52], while XGBoost 3.3.0 and LightGBM v4.7.0 were implemented using their optimised gradient boosting libraries [30,53]. Model performance was evaluated using the coefficient of determination (R^2^) and root mean squared error (RMSE):

(2) $${\;R}^{2\;}=1-\;\frac{\sum {\left(yi-\hat{yi}\right)}^{2}}{\sum {\left(yi-\bar{yi}\right)}^{2}}$$

(3) $$RMSE=\sqrt{\frac{1}{n}\sum {\left(yi-\hat{yi}\right)}^{2}}$$

Among all evaluated algorithms, LightGBM demonstrated the best predictive performance on the external scaffold-based test set and was, therefore, selected as the final MAPK3 QSAR model.

### 4.5. Cross-Validation and Applicability Domain Analysis

Five-fold cross-validation of the training data was used for internal validation [54]. Due to the small size and chemically diverse nature of the dataset, there was some variation in fold performance. The mean cross-validation R^2^ value was approximately 0.067, and the standard deviation was rather high, which led to moderate instability considering the limited variability of scaffolds and the small number of highly active compounds. This result is representative of a kinase-oriented QSAR with very strict scaffold splitting and is the result of the problems associated with extrapolation of activity to different chemical scaffolds [29,55].

The applicability domain (AD) analysis was performed using the Williams plot approach to evaluate prediction reliability and influential compounds. Leverage values were calculated according to Equation (4):

(4) $$h_{i\;=\;x_{i}^{T}{\left(X^{T}X\right)}^{-1}xi}$$

The warning leverage threshold was calculated using:

(5) $${\;h}^{*}=\;\frac{3\left(p+1\right)}{n}$$

where *p* = number of model descriptors and *n* = number of training compounds. For the final MAPK3 model: *n* = 725, *p* = 150, and h* = 0.6248.

Standardised residuals beyond ±3 were considered response outliers. According to the applicability domain analysis, 2.48% of the compounds were identified as residual outliers, 0.28% as high leverage compounds, and 0.14% as critical influential compounds. Based on these results, structural reliability and stability of the model are acceptable.

### 4.6. Classification-Oriented Validation, Permutation Feature Importance, and Hits Identification

The model was developed as a regression QSAR model, but further classification-based validation was done to assess the discriminatory power between the active and inactive compounds. Compounds with *pIC*_50_ > 6 were considered active, and compounds with *pIC*_50_ < 6 were considered inactive. The final model produced an area under the ROC curve (AUC) of 0.898, which showed excellent classification performance with good separation between the active and inactive MAPK3 inhibitors.

Permutation importance analysis was used to determine influential molecular features for model predictions. The feature importance was calculated by randomly permuting the individual features and calculating the impact on the predictive performance [32]. Several structural features derived from fingerprint contributions were found to be dominant, indicating that local substructural motifs play a strong role in the inhibitory activity of MAPK3. The importance distribution observed also indicates the presence of activity cliffs, where structurally related compounds show significant differences in potency [56]. Such behaviour is commonly observed in kinase inhibitor datasets and may partially explain the moderate external predictive performance observed under scaffold-based validation.

Finally, the full selected feature set was used to retrain the LightGBM model and rank the compounds based on the predicted *pIC*_50_ value. The highest-ranked compounds contained several experimental *pIC*_50_ values above 8.0, which are extremely potent inhibitors of MAPK3. Predicted activities showed a reasonable agreement with experimentally reported values, and the model was able to prioritise potent kinase inhibitors.

Final hit lists included: ChEMBL compound identifiers, canonical SMILES strings, experimental *pIC*_50_ values, and predicted *pIC*_50_ values. These compounds represent promising candidates for further kinase inhibitor optimisation and structure-based drug design studies targeting MAPK3.

### 4.7. Receptor Ligand Preparation, Molecular Docking Analysis, and ADMET Analysis

The three-dimensional (3D) structure of MAPK3 (PDB: 4QTB) was retrieved from the Protein Data Bank (PDB) [57]. The retrieved structure was cleaned with the help of *UCSF Chimaera v1.17.1* [57,58] by removing water molecules, crystallographic cofactors, and extra chains. Hydrogen atoms were then added to the protein structures, followed by energy minimisation to optimise their stability. The processed structures were saved in PDB format for further analysis. *BIOVIA Discovery Studio Software v 2026* [59] was also used to estimate the binding pocket in order to find possible docking sites. *PyRx software v 0.8* was used to prepare the top hit molecules in order to ensure their suitability for molecular docking studies. All ligands were first subjected to energy minimisation to maximise their structural conformations. The minimised ligands were subsequently converted to PDBQT format prior to docking. *PyRx software v 0.8* was used to perform molecular docking of the top-ranked compounds to assess their binding affinity with the target protein using the following grid dimensions: X: 33.3964, Y: 46.9664, and Z: 56.4603 [60]. The binding modes were visualised and interpreted after docking in *BIOVIA Discovery Studio v 2026* and *Chimaera X software v 1.12* to gain insight into receptor–ligand interactions [57]. ADME properties of the selected hit compounds were predicted using the *SwissADME* web server [61]. Moreover, toxicity analysis was performed using the *ProTox-II v 3.0* web server [62] to predict potential toxicological risks associated with the selected compounds.

Furthermore, the docking protocol was validated through redocking of the co-crystallised ligand from MAPK3, and RMSD analysis was performed to confirm the reliability of the docking methodology. In addition, known MAPK3 inhibitors, such as Ravoxertinib and Ulixertinib, were included as reference control compounds for comparative evaluation of binding interactions and docking performance.

### 4.8. Dft, Molecular Dynamics Simulation, and Mm-Gbsa Analysis

The selected drug candidates’ geometries were fully optimised using the *Gaussian 09 software v R.E.01* DFT/B3LYP [63] approach with multiple basis sets. The *Gauss View v 6* application for molecular visualisation was used to evaluate the Gaussian output files. In the gas phase, DFT-derived quantum chemical parameters were computed using the lowest unoccupied molecular orbital and highest occupied molecular orbital (HOMO–LUMO) energy values according to the numbering scheme allocated to the compounds [57]. Furthermore, the optimised structures’ coordinating groups’ effective charges, excitation energy, and oscillator strength were ascertained.

The stability of the docked complexes was studied through the MD simulation analysis. Top compounds identified through the molecular docking were subjected to the MD analysis. The *Desmond Module v 2026-2* of Schrödinger’s explicit solvent MD package, along with a fixed OPLS 2005 force field, was used for the MD simulation [57]. The protein preparation wizard was used to create a protein–ligand complex with a predetermined simple point charge (SPC) water model and an orthorhombic box shape (10 Å 10 Å 10 Å) [57]. To neutralise it, a NaCl solution was introduced to the system. The isosmotic condition of the simulation box remained intact through the addition of 0.15 m NaCl [64]. A specified equilibrium was achieved before the simulation’s production run. The modal system was submitted for simulation at 200 ns steps with the OPLS_2005 force field. A temperature of 300 K was maintained using the Nosé–Hoover chain thermostat algorithm, while a pressure of 1.01325 bar with isotropic coupling was maintained using the Martyna–Tobias–Klein barostat method [57,65]. All other parameters were maintained at their default values, with a Coulombic cutoff of 0.9 nm. Finally, the results of the MD simulation were examined for solvent accessible surface area (SASA), radius of gyration (Rg), root mean square deviation (RMSD), root mean square fluctuation (RMSF), etc., using the simulation interaction diagram (SID) tool.

Additionally, MD simulations and MM-GBSA binding free energy calculations (200 ns) were also performed for the co-crystallised ligand and reference inhibitors Ravoxertinib and Ulixertinib to provide a direct comparative framework for evaluating the dynamic stability and energetic behaviour of the proposed MAPK3 inhibitors.

The binding free energies (Δ*G* bind) of the chosen protein–ligand complexes were determined using the MM-GBSA method and the default settings of the prime *MM-GBSA module* in *Schrödinger software v 2026-2* [57,65]. The *Glide pose viewer* file was used for the analysis, and MM-GBSA computations were used to determine the relative binding affinity of ligands to the receptor, which was expressed in kcal/mol. Stronger binding affinity is indicated by larger negative binding energies, which are estimates of the free binding energies.

## 5. Conclusions

In this study, a computational framework based on integrated artificial intelligence was developed to identify potential MAPK3 inhibitory compounds for oral inflammatory and proliferative diseases through a combination of QSAR modelling, machine learning, molecular docking, DFT calculations, molecular dynamics simulations, and MM-GBSA molecular mechanics analysis. The LightGBM model exhibited high predictive capability and was able to prioritise highly active compounds. Based on the molecular docking and simulation studies, compounds 58324148 and 137531515 were found to be computationally prioritised MAPK3 binding compounds with good binding affinity, structural stability, and energetics, and compound 58324148 showed a better overall stability and interaction behaviour. Despite these computational observations, the study remains limited by the absence of experimental validation. Thus, future studies are needed for in vitro and in vivo validation, kinase selectivity profiling, ADMET evaluation, and lead optimisation to validate the biological relevance of the compounds in oral diseases related to MAPK3, such as oral squamous cell carcinoma, periodontitis, and chronic inflammatory oral disorders.

## Data Availability

The data supporting the findings of this study are available from the corresponding author upon reasonable request.

## Supplementary Materials

The following supporting information can be downloaded at: https://www.mdpi.com/article/10.3390/ph19081309/s1, Figure S1. Dynamic correlation, conformational stability and MM-GBSA analysis of MAPK3 complexes. (A) Dynamic cross-correlation matrix (DCCM) of the MAPK3–58324148 complex showing correlated and anti-correlated residue motions. (B) Free energy landscape (FEL) analysis of the MAPK3–58324148 complex showing conformational stability and low-energy states. (C) Rg analysis of the MAPK3–58324148 complex for the duration of the simulation. (D) Dynamic cross-correlation matrix (DCCM) of the MAPK3–137531515 complex. (E) Free energy landscape (FEL) analysis of the MAPK3–137531515 complex. (F) Radius of gyration (Rg) analysis of the MAPK3–137531515 complex during simulation. (G) MM-GBSA energy decomposition analysis of the MAPK3–58324148 complex showing energetic contributions over simulation time. (H) MM-GBSA energy decomposition analysis of the MAPK3–137531515 complex. Figure S2. Validation of MAPK3 docking protocol and comparative interaction analysis. (A) Co-crystallized ligand re-docked into the MAPK3 binding pocket showing conserved interaction patterns. (B) Binding interaction profile of Ulixertinib (reference MAPK3 inhibitor). (C) Binding interaction profile of Ravoxertinib (reference MAPK3 inhibitor). (D) Comparative binding affinity heatmap of co-crystal, Ulixertinib, and Ravoxertinib against MAPK3. Hydrogen bonds, hydrophobic interactions, and electrostatic contacts are shown to evaluate docking consistency and benchmark the binding behavior of the proposed compounds. Figure S3. Comparative ADMET and toxicity radar profiling of the co-crystal, reference control drugs (Ulixertinib and Ravoxertinib), and compounds (58324148 and 137351515), highlighting physicochemical drug-likeness and predicted safety liabilities across multiple endpoints. Figure S4. Molecular dynamics simulation analysis of MAPK3 complexes. (A) Root mean square deviation (RMSD) profiles of MAPK3 bound to co-crystal, Ulixertinib, and Ravoxertinib over 200 ns simulation time. (B) Root mean square fluctuation (RMSF) analysis showing residue-level flexibility of MAPK3 in complex with co-crystal, Ulixertinib, and Ravoxertinib. (C) Hydrogen bond occupancy analysis illustrating the time-dependent stability of intermolecular interactions between MAPK3 and co-crystal, Ulixertinib, and Ravoxertinib during the simulation period. Figure S5. Advanced molecular dynamics and conformational analysis of MAPK3–ligand complexes. Panels (A–D,M) represent the MAPK3–co-crystal complex, panels (E–H,N) represent the MAPK3–Ulixertinib complex, and panels (I–L,O) represent the MAPK3–Ravoxertinib complex. (A,E,I) Free energy landscape (FEL) based on principal component analysis showing conformational minima. (B,F,J) Dynamic cross-correlation matrix (DCCM) indicating residue–residue correlated and anti-correlated motions. (C,G,K) Principal component analysis (PCA) conformational sampling of protein dynamics. (D,H,L) Probability density function (PDF) of dominant conformational states. (M–O) Radius of gyration (Rg) profiles showing structural compactness and stability over time. Figure S6. MM-GBSA binding energy decomposition analysis of MAPK3–ligand complexes over 200 ns simulation. (A) Energy components for MAPK3–co-crystal complex. (B) Energy components for MAPK3–Ulixertinib complex. (C) Energy components for MAPK3–Ravoxertinib complex. The plots show time-dependent variation of total binding free energy (ΔG_bind) and its contributing terms, including van der Waals, electrostatic (Coulomb), hydrogen bonding, and solvation energy components. Co-crystal (A) is shown in black, Ulixertinib (B) in blue, and Ravoxertinib (C) in red/orange/purple as per trajectory-specific energy terms. Table S1: The top 20 compounds identified by the LightGBM model.
