# Supplementary material for: Integrated AI-Driven Discovery of MAPK3 Inhibitors for Oral Inflammatory and Proliferative Diseases
Source: Pharmaceuticals (Basel). 2026 Aug 19;19(8):1309. doi: 10.3390/ph19081309 (PMC13516356; doi:10.3390/ph19081309)
Supplement: Supplementary file 1 [file pharmaceuticals-19-01309-s001.zip › Supplementary File S2.pdf]

Integrated AI-Driven Discovery of MAPK3 Inhibitors for Oral Inflammatory and Proliferative Diseases

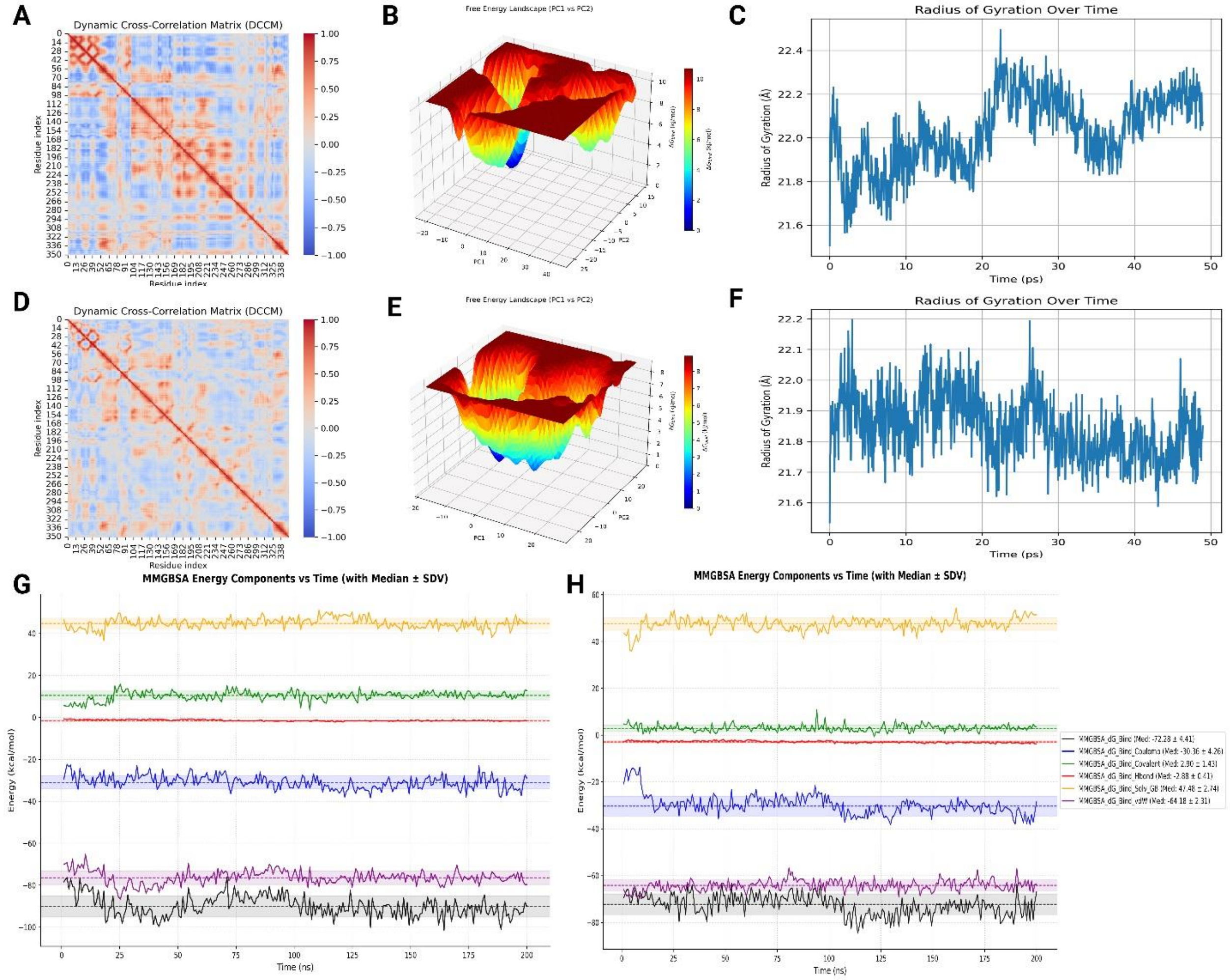

Figure S1. Figure S1. Dynamic correlation, conformational stability and MM-GBSA analysis of MAPK3 complexes. (A) Dynamic cross-correlation matrix (DCCM) of the MAPK3–58324148 complex showing correlated and anti-correlated residue motions. (B) Free energy landscape (FEL) analysis of the MAPK3–58324148 complex showing conformational stability and low-energy states. (C) Rg analysis of the MAPK3–58324148 complex for the duration of the simulation. (D) Dynamic cross-correlation matrix (DCCM) of the MAPK3–137531515 complex. (E) Free energy landscape (FEL) analysis of the MAPK3–137531515 complex. (F) Radius of gyration (Rg) analysis of the MAPK3–137531515 complex during simulation. (G) MM-GBSA energy decomposition analysis of the MAPK3–58324148 complex showing energetic contributions over

simulation time. (H) MM-GBSA energy decomposition analysis of the MAPK3–137531515 complex.

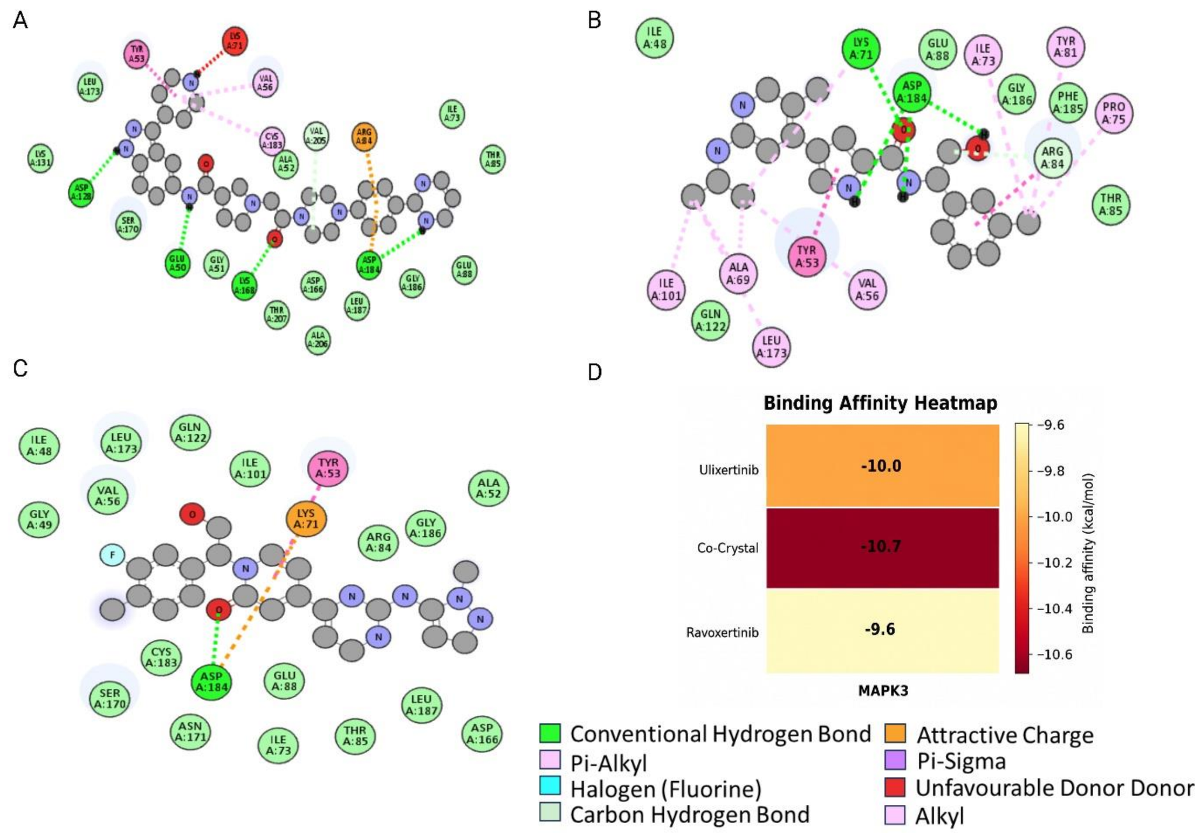

Figure S2. Validation of MAPK3 docking protocol and comparative interaction analysis. (A) Co-crystallized ligand re-docked into the MAPK3 binding pocket showing conserved interaction patterns. (B) Binding interaction profile of Ulixertinib (reference MAPK3 inhibitor). (C) Binding interaction profile of Ravoxertinib (reference MAPK3 inhibitor). (D) Comparative binding affinity heatmap of co-crystal, Ulixertinib, and Ravoxertinib against MAPK3. Hydrogen bonds, hydrophobic interactions, and electrostatic contacts are shown to evaluate docking consistency and benchmark the binding behavior of the proposed compounds.

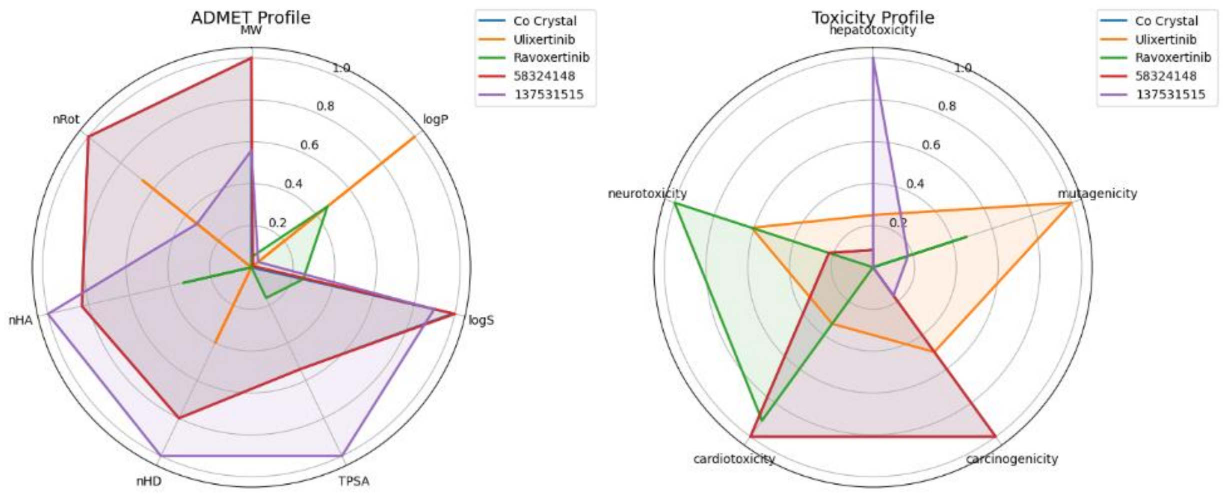

Figure S3. Comparative ADMET and toxicity radar profiling of the co-crystal, reference control drugs (Ulixertinib and Ravoxertinib), and compounds (58324148 and 137351515), highlighting physicochemical drug-likeness and predicted safety liabilities across multiple endpoints.

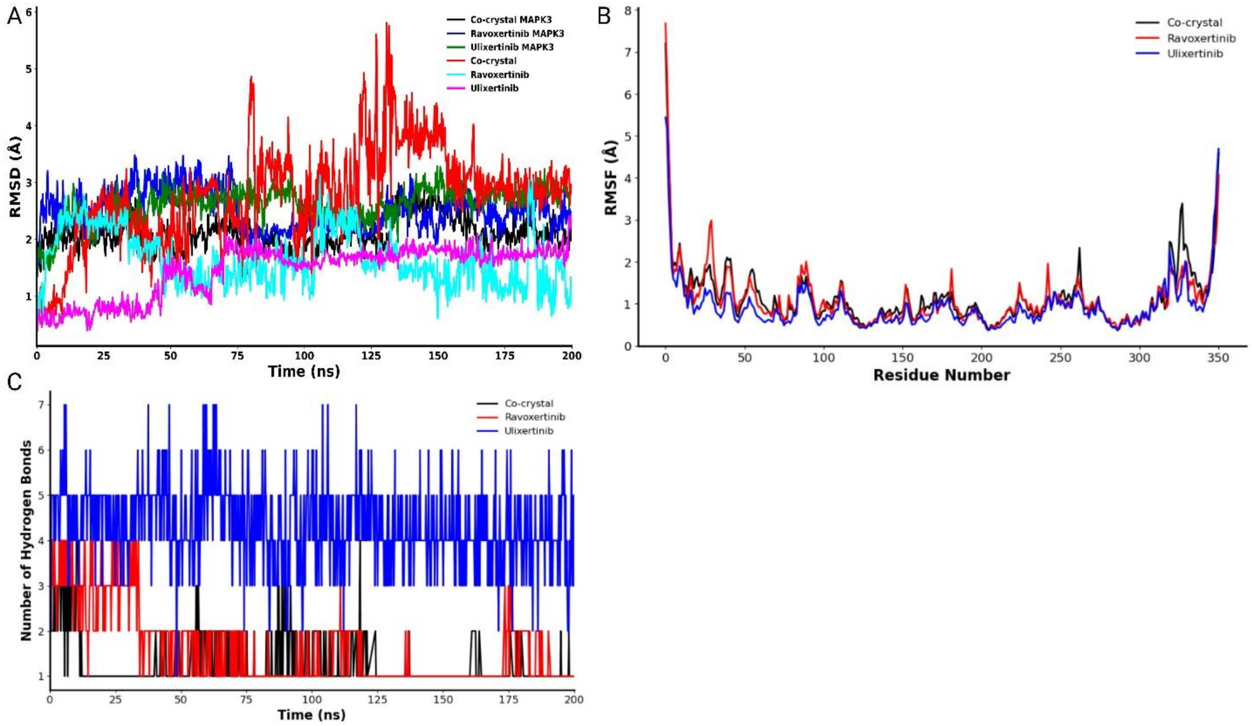

Figure S4. Molecular dynamics simulation analysis of MAPK3 complexes. (A) Root mean square deviation (RMSD) profiles of MAPK3 bound to co-crystal, Ulixertinib, and Ravoxertinib over 200 ns simulation time. (B) Root mean square fluctuation (RMSF) analysis showing residue-level flexibility of MAPK3 in complex with co-crystal, Ulixertinib, and Ravoxertinib. (C)

Hydrogen bond occupancy analysis illustrating the time-dependent stability of intermolecular interactions between MAPK3 and co-crystal, Ulixertinib, and Ravoxertinib during the simulation period.

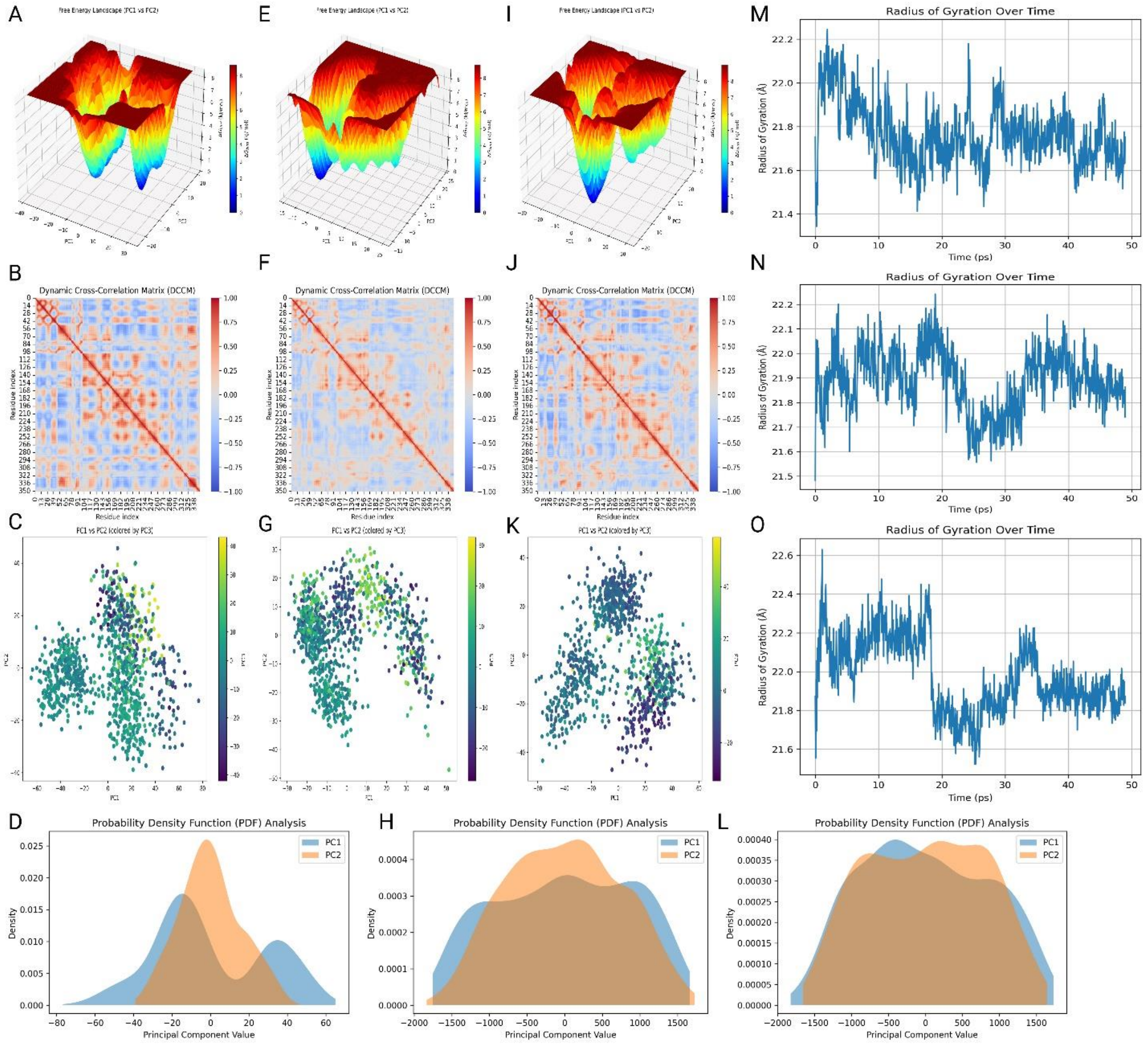

Figure S5. Advanced molecular dynamics and conformational analysis of MAPK3–ligand complexes. Panels A, B, C, D, and M represent the MAPK3–co-crystal complex, panels E, F, G, H, and N represent the MAPK3–Ulixertinib complex, and panels I, J, K, L, and O represent the MAPK3–Ravoxertinib complex. (A, E, I) Free energy landscape (FEL) based on principal component analysis showing conformational minima. (B, F, J) Dynamic cross-correlation matrix (DCCM) indicating residue–residue correlated and anti-correlated motions. (C, G, K) Principal

component analysis (PCA) conformational sampling of protein dynamics. (D, H, L) Probability density function (PDF) of dominant conformational states. (M, N, O) Radius of gyration (Rg) profiles showing structural compactness and stability over time.

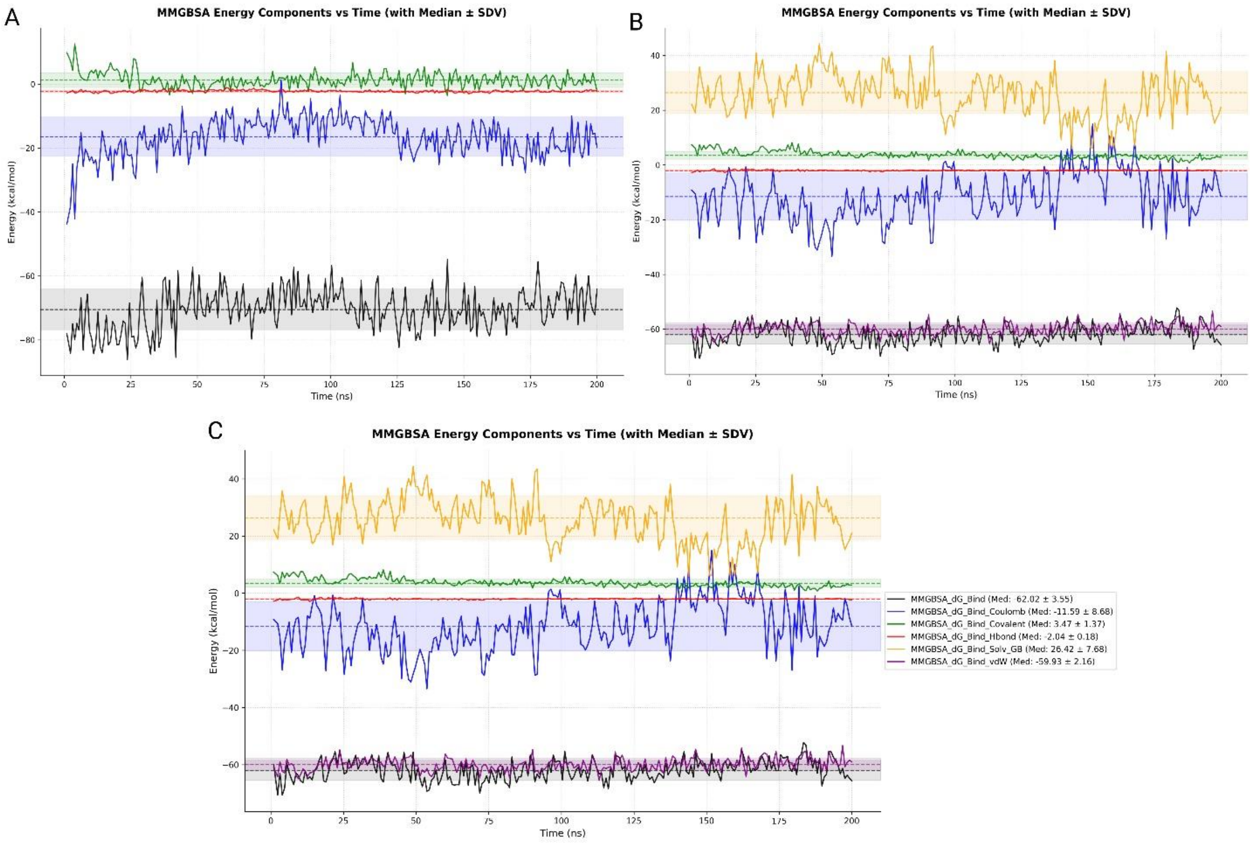

Figure S6. MM-GBSA binding energy decomposition analysis of MAPK3–ligand complexes over 200 ns simulation. (A) Energy components for MAPK3–co-crystal complex. (B) Energy components for MAPK3–Ulixertinib complex. (C) Energy components for MAPK3–Ravoxertinib complex. The plots show time-dependent variation of total binding free energy (ΔG_bind) and its contributing terms, including van der Waals, electrostatic (Coulomb), hydrogen bonding, and solvation energy components. Co-crystal (A) is shown in black, Ulixertinib (B) in blue, and Ravoxertinib (C) in red/orange/purple as per trajectory-specific energy terms.
